# Clinical Validation of a Rapid Variant-Proof RT-RPA Assay for the Detection of SARS-CoV-2

**DOI:** 10.3390/diagnostics12051263

**Published:** 2022-05-19

**Authors:** Dounia Cherkaoui, Judith Heaney, Da Huang, Matthew Byott, Benjamin S. Miller, Eleni Nastouli, Rachel A. McKendry

**Affiliations:** 1 London Centre for Nanotechnology, University College London, London WC1E 6BT, UK; dounia.cherkaoui.17@ucl.ac.uk (D.C.); d.huang@ucl.ac.uk (D.H.); ben.miller.13@ucl.ac.uk (B.S.M.); 2 Division of Medicine, University College London, London WC1E 6BT, UK; 3 Advanced Pathogen Diagnostics Unit, University College London Hospitals (UCLH) NHS Trust, London NW1 2BU, UK; jude.heaney@hslpathology.com (J.H.); matt.byott@nhs.net (M.B.); 4 Infection, Immunity and Inflammation Department, Great Ormond Street Institute for Child Health (ICH), University College London, London WC1N 1EH, UK

**Keywords:** SARS-CoV-2, isothermal amplification, recombinase polymerase amplification, clinical samples

## Abstract

The COVID-19 pandemic has unveiled a pressing need to expand the diagnostic landscape to permit high-volume testing in peak demand. Rapid nucleic acid testing based on isothermal amplification is a viable alternative to real-time reverse transcription polymerase chain reaction (RT-PCR) and can help close this gap. With the emergence of SARS-CoV-2 variants of concern, clinical validation of rapid molecular tests needs to demonstrate their ability to detect known variants, an essential requirement for a robust pan-SARS-CoV-2 assay. To date, there has been no clinical validation of reverse transcription recombinase polymerase amplification (RT-RPA) assays for SARS-CoV-2 variants. We performed a clinical validation of a one-pot multi-gene RT-RPA assay with the E and RdRP genes of SARS-CoV-2 as targets. The assay was validated with 91 nasopharyngeal samples, with a full range of viral loads, collected at University College London Hospitals. Moreover, the assay was tested with previously sequenced clinical samples, including eleven lineages of SARS-CoV-2. The rapid (20 min) RT-RPA assay showed high sensitivity and specificity, equal to 96% and 97%, respectively, compared to gold standard real-time RT-PCR. The assay did not show cross-reactivity with the panel of respiratory pathogens tested. We also report on a semi-quantitative analysis of the RT-RPA results with correlation to viral load equivalents. Furthermore, the assay could detect all eleven SARS-CoV-2 lineages tested, including four variants of concern (Alpha, Beta, Delta, and Omicron). This variant-proof SARS-CoV-2 assay offers a significantly faster and simpler alternative to RT-PCR, delivering sensitive and specific results with clinical samples.

## 1. Introduction

Severe acute respiratory syndrome coronavirus 2 (SARS-CoV-2), the novel coronavirus responsible for the COVID-19 pandemic, has had a devastating impact worldwide, infecting more than 520 million people and causing more than 6.2 million deaths [1].

Access to quality and affordable diagnostic tests is still limited in many countries, and even countries with wider access have struggled to meet the high-volume demand at the peak of the pandemic [2]. Daily numbers of SARS-CoV-2 tests per country per capita vary widely across nations [3]. There is a pressing need to widen access to diagnostics, therapeutics, and vaccines for all nations [4]. For this reason, global initiatives such as COVAX [5] and The Access to COVID-19 Tools Accelerator [6] aim to achieve fairer access to vaccines and diagnostics in low- and middle-income countries.

The gold standard diagnostic for SARS-CoV-2 remains nucleic acid amplification testing, with real-time reverse transcription polymerase chain reaction (RT-PCR) the most widely used method [7], however, it is not suited to all settings. RT-PCR is based on nucleic acid amplification using thermocycling which typically requires an expensive instrument, and a reliable power supply and takes between 45 to 90 min [8].

Several rapid nucleic acid tests for SARS-CoV-2 have been developed and successfully validated with clinical samples, especially assays that capitalise on fast and isothermal amplification, in particular recombinase polymerase amplification (RPA) and loop-mediated isothermal amplification (LAMP) [9,10,11,12,13,14,15]. RPA typically amplifies at low temperatures (37–39 °C) that can be reached and maintained by very simple and inexpensive methods (e.g., water bath, hand warmer bag, body temperature) [16], compared to LAMP which requires a heating device maintained at 62 °C [9,10]. This makes RPA a better candidate for low-resource settings. While rapid antigen tests can detect infectious cases and help close the gap to accessible testing, they have lower sensitivity compared to RT-PCR (as low as 40% sensitivity when looking at symptom-free individuals) [17]. It has been argued that the two types of tests are not comparable as they do not detect the same type of viral material [18].

A range of SARS-CoV-2 RT-RPA assays have been reported in the literature [11,12,13,14], however, unlike RT-PCR, they do not typically target multiple SARS-CoV-2 genes, risking a loss of sensitivity and specificity. Moreover, they often require multiple steps, thus, adding complexity and delays. We recently reported a proof-of-concept multiplexed, 1–2 step, fast (20–30 min) RT-RPA SARS-CoV-2 assay to simultaneously detect two conserved targets: the envelope protein (E) and RNA-dependent RNA polymerase (RdRP) genes [16]. The agile multi-gene platform offers two complementary detection methods: real-time fluorescence or dipstick. Of the two methods, the fluorescence assay was found to be the most sensitive. The analytical sensitivity of the fluorescence test was evaluated using synthetic RNA and found to be 9.5 (95% CI: 7.0–18) RNA copies per reaction for the E gene and 17 (95% CI: 11–93) RNA copies per reaction for the RdRP gene. No cross-reactivity was found with common seasonal coronaviruses, SARS-CoV, and MERS-CoV model samples. However, in the previous study, it was not clinically validated or benchmarked to RT-PCR, to achieve quantitative detection, nor evaluated with clinical variants of concern.

In the literature, obtaining quantitative or semi-quantitative results with RPA assays has been challenging [19]. Quantification of results for molecular diagnostic tests can be useful, as it can provide information with clinical relevance for patients [20]. For viral infections such as COVID-19, knowing the viral load, which is the amount of measurable virus in a sample, may help assess the risk of transmission [21], estimate the temporal dynamic of the infection [22], and potentially predict the severity and mortality [23,24]. The quantification cycle (Cq) values from RT-PCR experiments have been extensively used as a surrogate for viral load [25].

Moreover, RT-PCR assays for SARS-CoV-2 typically target multiple regions or genes to mitigate the effect of viral mutations [26]. Genetic SARS-CoV-2 lineages have been emerging in different geographical locations and comprise key mutations [27]. Genetic lineages are monitored through epidemiological and genomic surveillance [28], but only in some countries, as this requires extensive laboratory capacity, expertise, and funds [29].

Variants of concern (VOCs) are SARS-CoV-2 variants with mutations that have a predicted impact on transmissibility, severity of the disease, and effectiveness of pharmaceutical interventions and vaccines [27]. To date, five VOCs have been identified, namely the Alpha (lineage B.1.1.7), Beta (lineage B.1.351), Delta (lineage B.1.617.2.), Gamma (lineage P.1), and Omicron (B.1.1.529) variants.

Sequencing results are necessary to confirm VOCs and enhance surveillance of new emerging variants [27,28]. However, routine diagnostic assays should be unimpacted by mutations and be able to detect all variants [26]. This is particularly crucial in areas of the world where sequencing capacities are limited or non-existent, as the emergence of a new variant can also escape diagnostic tests. In this case, patients infected by this new undetected variant could appear negative for SARS-CoV-2, spreading the variant further.

With the rapid emergence of new variants, all molecular assays for SARS-CoV-2 should be robust to genetic variations of the virus [26]. While clinical validation of isothermal assays for SARS-CoV-2 has failed to report on their ability to detect different lineages and VOCs [9,10,11,12,13,14,15], this is a critical requirement to prove they are suitable alternatives to RT-PCR.

**Figure 1:**
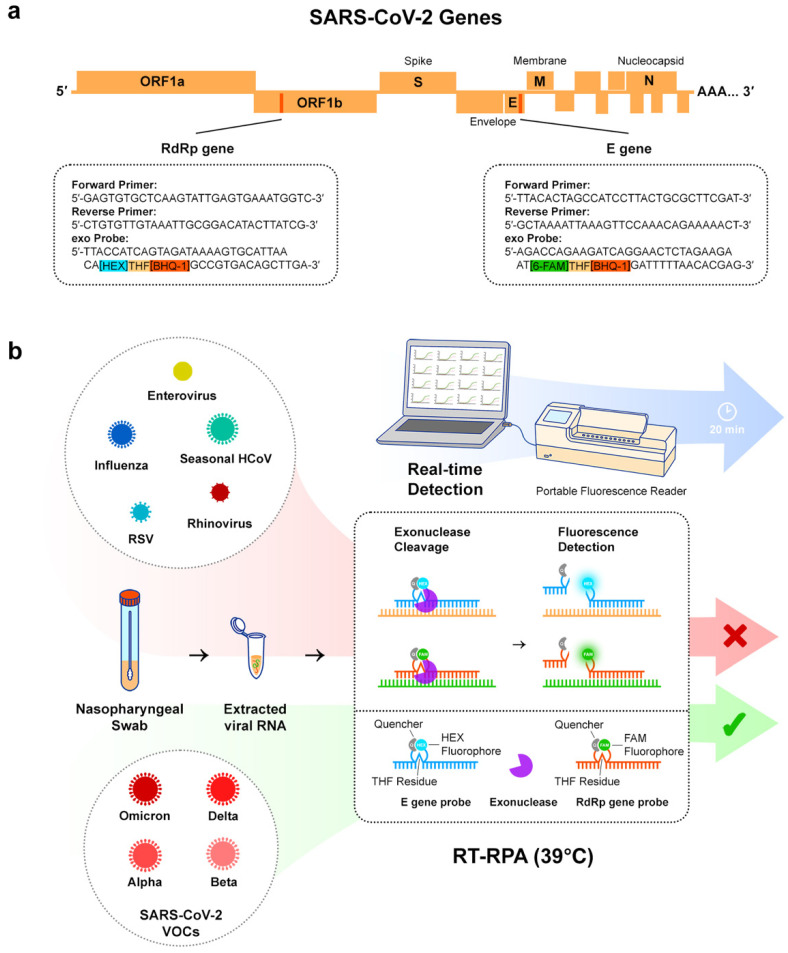
Schematic illustration of the one-pot RT-RPA multi-gene assay. (**a**) Representation of SARS-CoV-2 genes and sequences of the primers and exo probes used for the E and RdRP gene targets. (**b**) Nasopharyngeal swabs from patients were tested. After extraction of viral RNA from the swabs, the samples were screened with the multi-gene RT-RPA assay (isothermal amplification at 39 °C) with an amplification time of 20 min. The validation panel included SARS-CoV-2 variants of concern (VOCs) and other respiratory pathogens.

Herein, we report the clinical validation of the real-time fluorescence RT-RPA assay with 91 RNA extracts from nasopharyngeal swabs collected at University College London Hospitals (Figure 1). The work builds on our previously published research of the first one-pot multi-gene RT-RPA assay to detect SARS-CoV-2 RNA, targeting the E and RdRP genes [16]. We demonstrated the ability of the assay to detect eleven SARS-CoV-2 lineages, including Alpha, Beta, Delta, and Omicron. We also investigated a semi-quantitative method to translate the time to the threshold (TT) from RT-RPA results into viral load equivalents.

## 2. Methods

### 2.1. Clinical Samples

The RT-RPA assay was validated with 91 residual de-identified RNA extracts from nasopharyngeal swabs collected at University College London Hospitals. The clinical samples were collected from suspected COVID-19 patients during a medical examination as part of the standard of care. The residual material was used for clinical validation of the developed RT-RPA assay. Clinical samples were selected for RT-RPA analysis based on the SARS-CoV-2 lineages revealed from whole genome sequencing. In addition, a range of Cq values were included. Although these samples were not routinely screened to eliminate the presence of other respiratory pathogens, the real-time RT-PCR assay has previously been shown not to cross-react [30].

The RNA was extracted on the QiaSymphony platform using the DSP virus pathogen mini extraction kit from Qiagen. Extraction was performed in 200 µL of viral transport media (VTM), 200 µL of lysis buffer was added, including the additional lysis step with ATL buffer, and finally, samples were eluted in 60 µL elution buffer.

A total of 55 positive and 36 negative samples were tested (blinded). Sensitivity and specificity were calculated using the statistical calculator for clinical research VassarStats (http://vassarstats.net/, accessed on 6 May 2022).

### 2.2. Real-Time RT-PCR

All samples were initially screened at University College London Hospitals by real-time RT-PCR targeting the N gene and using the extraction-free SARS-CoV-2 diagnosis method [30]. In brief, 2 µL of sample in VTM was added to the RT-PCR mix comprised of 5 μL of 4X TaqMan Fast Virus 1-step Master Mix (Applied Biosystems, Waltham, MA, USA), SARS-CoV-2 N gene primers, RNase P primers and probes each at a final concentration of 250 nM and the reaction volume made up to 20 µL with nuclease-free water. Thermocycling was performed at 56 °C for 15 min for reverse transcription, followed by 95 °C for 20 s and then 45 cycles at 95 °C for 3 s, 60 °C for 30 s using an Applied Biosystems QuantStudio 5 real-time PCR system, and Cq values were determined using QuantStudio Design and Analysis Software (Thermo Fisher Scientific, Waltham, MA, USA).

Confirmatory real-time RT-PCR for presumptive positive samples was performed at UCL with the N1 gene (US CDC) protocol [31]. The TaqMan Fast Virus 1-Step MasterMix (Thermo Fisher Scientific) and QuantStudio 5 real-time PCR system were used. The positivity criterion was Cq < 40.

### 2.3. Sequencing

Total nucleic acid extractions were performed on the QiaSymphony using the DSP virus pathogen extraction kit (Qiagen, Hilden, Germany). SARS-CoV-2 full genome amplification was performed in accordance with protocols published by the ARTIC network [32] using the ARTIC primer set from Integrated DNA Technologies (Coralville, IA, USA) to create tiled amplicons across the viral genome. Libraries were prepared using the Illumina DNA prep kit and sequencing on Illumina platforms (Illumina, San Diego, CA, USA). Genomes were either assembled in-house using the V3 ARTIC network Illumina bioinformatics pipeline or by PHE/UKHSA using the ARTIC V4 version and made available in the COG-UK database [33]. Pangolin [34] was used to assign an epidemiological lineage to each sequence and mutations were analysed using COG-UK Mutation Explorer [35].

### 2.4. Multi-Gene RT-RPA

RPA primers and exo probes (Figure 1a) were designed and evaluated in our previous published work [16]. Primers were ordered from Integrated DNA Technologies and probes were synthesised by Eurogentec.

Each RT-RPA reaction was prepared as a 50 µL reaction and contained a TwistAmp^®^ exo RPA pellet (TwistDX) resuspended in 29.5 µL Rehydration Buffer, 2.1 µL of both forward and both reverse primers (at a concentration of 10 µM), 0.6 µL of both exo probes, 2.5 µL of M-MLV reverse transcriptase (Thermo Fisher Scientific), 0.9 µL of nuclease-free water, and 5 µL of RNA extract from clinical samples. For each RT-RPA run, a non-template control (nuclease-free water added instead of the sample) was included for quality control. Finally, a magnetic bead was added to the tube and 2.5 µL of magnesium acetate (at a concentration of 280 mM) was added to the lid (magnesium acetate is required to start the RPA reaction). The tubes were spun to start the reactions. The tubes were incubated at 39 °C for 20 min with magnetic shaking and the fluorescence was measured in real-time using the T16-ISO multi-channel portable fluorescence reader (Axxin, Fairfield, VIC, Australia) (Figure 1b).

Background subtraction was applied to the fluorescence signals by subtracting all values after t = 3 min by the fluorescence signal before that, and by setting all values before t = 3 min to zero. This method aims to normalise all runs and minimise artefacts produced by the mixing during the first few minutes which can alter the fluorescence reading at the beginning of the assay.

The individual fluorescence thresholds for the E gene and RdRP gene, equal to 112 and 13, respectively, were previously determined [16]. These thresholds were used to assess if a sample was positive for each gene (fluorescence signal above the threshold at t = 20 min) or negative (fluorescence signal below the threshold at t = 20 min).

If a sample was negative for one gene but positive for the other, it was designated as ‘presumptive positive’ and required confirmatory RT-PCR to confirm the result.

### 2.5. Correlation between Cq and TT

The TT was calculated separately for E and RdRP genes for all true positive samples. The TT was defined as the time at which the fluorescence signal becomes higher than the fluorescence threshold.

The linear regression and Pearson correlation coefficient between Cq and TT values were plotted using Prism 9.

## 3. Results

### 3.1. Clinical Sensitivity and Specificity

A total of 91 RNA extracts from nasopharyngeal swabs were used for the clinical validation of the RT-RPA assay. The clinical samples provided by University College London Hospitals were previously confirmed negative or positive for SARS-CoV-2, using an RT-PCR standard of care assay. The Cq value was provided for each positive sample, to ensure a full range of Cq values (used as a proxy to infer viral load) was included in the clinical validation. Furthermore, these clinical samples were all sequenced to determine their SARS-CoV-2 lineage. Pathogen, lineage, Cq, and results for the samples of the validation panel can be found in Supplementary Table S1. The result of the clinical validation with the 91 samples is presented in Table 1.

Among the 55 positive samples, the RT-RPA assay was able to detect 53 samples correctly (among which two samples were initially detected as presumptive positive results and later confirmed by RT-PCR), resulting in clinical sensitivity of 96% (95% CI: 86–99). The two false negative results had relatively high Cq values of 32.0 and 37.3, respectively, reflecting very low viral loads which may not be consistently detected by RT-RPA. In the instance where only one gene target was detected, the samples were considered ‘presumptive positive’, which usually requires confirmation by another assay (e.g., real-time RT-PCR) or by re-screening the sample by RT-RPA if a confirmatory RT-PCR is not available. A total of four samples were identified as presumptive positive. We decided to confirm these clinical samples by real-time RT-PCR (Supplementary Table S2). Interestingly, the RT-RPA assay showed it was able to detect many samples with high Cq values (associated with a low viral load), up to Cq equal to 35.0.

All negative samples for SARS-CoV-2 in the validation panel were clinical samples confirmed for other respiratory pathogens, namely human seasonal coronaviruses (HCoV-NL63, HCoV-OC43, HCoV-229E, HCoV-HKU1), influenza (Flu) A and B, rhinovirus, enterovirus, and respiratory syncytial virus (RSV), shown in Supplementary Table S1.

Only one false positive was obtained using the RT-RPA with detection of both E and RdRP genes, resulting in clinical specificity of 97% (95% CI: 84–100). This sample was previously confirmed positive for HCoV-NL63 and negative for SARS-CoV-2. We considered the possibility of cross-reactivity of the assay with HCoV-NL63, a seasonal coronavirus also classified as a ‘common cold’ coronavirus. Further bioinformatics analysis of the RPA primers and probes confirmed the significant variations (≤67% identity) between the genomes of SARS-CoV-2 and HCoV-NL63 in the targeted regions (Supplementary Figure S1). In addition, we previously demonstrated the high specificity of the RT-RPA assay, showing no cross-reactivity with other coronaviruses (including HCoV-NL63) when using RNA model samples [16]. To confirm this false positive was not due to cross-reactivity, we re-screened this sample three times by RT-RPA and consecutively found that it was negative (Supplementary Figure S2). We concluded that the RT-RPA assay detected SARS-CoV-2 specifically and showed no cross-reactivity with the respiratory pathogens tested.

We also calculated the positive and negative predictive values (PPV and NPV) which were 98% (95% CI: 89–100) and 95% (95% CI: 80–99), respectively.

### 3.2. Detection of SARS-CoV-2 Variants

Several SARS-CoV-2 lineages were tested with the RT-RPA assay, including four VOCs, namely the Alpha, Beta, Delta, and Omicron variants (Supplementary Table S1). Sensitivity was calculated for each VOC (Table 2). The RT-RPA assay was able to detect 100% of the Beta, Delta, and Omicron samples. Only two out of fifteen Alpha samples were not detected, resulting in a sensitivity equal to 87%. The lower sensitivity for the Alpha samples may be explained by the relatively high Cq values of these two false negative samples (32.0 and 37.3). We also note that the number of samples for each VOC was unequal due to limited access to extracted clinical samples during the pandemic. Only a low number of Beta samples could be tested as Beta was less prevalent in patients in the UK.

**Figure 2:**
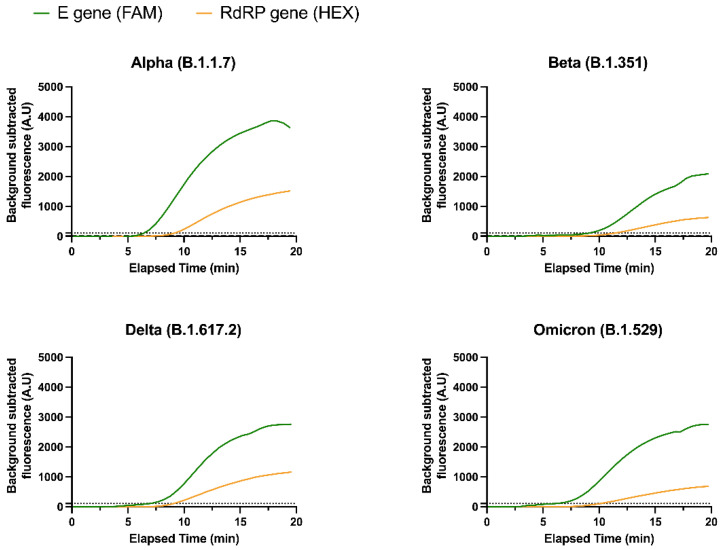
Detection of SARS-CoV-2 VOCs clinical samples by one-pot multi-gene RT-RPA real-time fluorescence assay. Detection of the four VOCs tested (Alpha, Beta, Delta, and Omicron) by both E and RdRP gene targets of the RT-RPA assay. Note: the Alpha sample is sample 8; the Beta sample is sample 55; the Delta sample is sample 70; the Omicron sample is sample 54.

Importantly, both gene targets were able to detect the four VOCs tested, showing that the multi-gene assay was exhibiting adequate plasticity to mutations present in VOCs (Figure 2). We observed a slightly lower FAM fluorescence signal for the Beta variant compared to other lineages. Bioinformatics analysis revealed that a characteristic single nucleotide polymorphism (SNP) of the Beta variant (nucleotide 26,456; C to T) affected the E gene exo probe, likely explaining the lower fluorescence signal for this target. The only other VOC mutation found in the target regions was a distinctive SNP of the Delta variant (nucleotide 15,451; G to A) in the RdRP gene; yet, neither the RdRP primers nor the exo probe were affected by this SNP, which explains why both sensitivity and fluorescence signal remained high for Delta samples.

### 3.3. Correlation between Cq and TT

**Figure 3:**
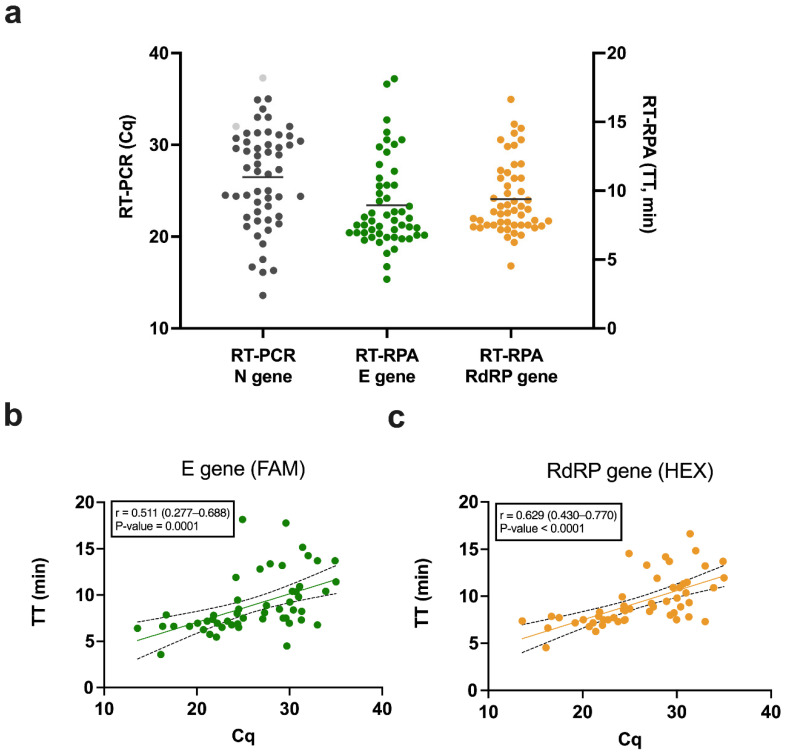
Correlation between quantification cycle (Cq) values from RT-PCR and time to threshold (TT) from RT-RPA. (**a**) Comparison of Cq from RT-PCR and TT from RT-RPA for all positive samples. Dark grey dots represent the true positive samples and light grey dots represent the false negative samples. The horizontal lines represent the mean Cq for RT-PCR and the mean TT for RT-RPA. (**b**) Linear regression between Cq values (RT-PCR N gene) and TT values (RT-RPA E gene) with the associated Pearson correlation coefficient r (95% CI) and *p*-value. The dashed lines represent the 95% confidence interval. The green dots represent the clinical samples detected by the RT-RPA E gene target. (**c**) Linear regression between Cq values (RT-PCR N gene) and TT values (RT-RPA RdRP gene) with the associated Pearson correlation coefficient r (95% CI) and *p*-value. The dashed lines represent the 95% confidence interval. The yellow dots represent the clinical samples detected by the RT-RPA RdRP gene target.

The Cq values from the RT-PCR N gene and the TT values from RT-RPA E and RdRP genes for all SARS-CoV-2 positive samples are shown in Figure 3a. The mean Cq was 26.5 and the mean TT was similar for both genes, at around 9 min. For all true positive results, the simple linear regression between Cq (ranging from 13.6 to 35.0) and TT values (ranging from 3 to 18 min) were plotted for both gene targets (Figure 3b,c). The Pearson correlation coefficients were 0.511 (95% CI: 0.277–0.688) and 0.629 (95% CI: 0.430–0.770) for the E and RdRP genes, respectively (Supplementary Table S3). The positive association of the TT values for the RdRP gene with Cq values was stronger than for the E gene. The correlation was statistically significant for both genes, with a *p*-value = 0.0001 for the E gene and a *p*-value < 0.0001 for the RdRP gene, hence, the correlation analysis of both genes is important to build evidence of such association and to what degree. Similar to using Cq values as a proxy for viral loads, we showed that the TT from RT-RPA runs may be used to deliver semi-quantitative results. However, we note that the variance was relatively high, which may limit the precision of the method.

## 4. Discussion

This clinical validation of the one-pot multi-gene RT-RPA assay with clinical demonstrated 96% sensitivity and 97% specificity in 91 nasopharyngeal swabs. Notably, this clinical sensitivity of the assay was above 90%, which is the ‘desirable sensitivity’ threshold set by the World Health Organisation [36]. The clinical specificity of the assay exactly matched the World Health Organisation acceptable requirements (≥97%). We believe the specificity results reported here were particularly relevant as the assay showed no cross-reactivity with the respiratory pathogens included as negative samples in the validation panel. After confirming all presumptive positive results with RT-PCR results, we demonstrated that the only false positive which occurred was unlikely caused by cross-reactivity, as corroborated by a previous specificity study with model samples [16], bioinformatics analysis, and by confirming a negative result when the sample was re-run three additional times. For these reasons, we are inclined to think this false positive was not due to an inherent design issue or cross-reactivity with seasonal coronaviruses but possibly due to experimental error while performing the blind screen. Mitigation strategies to prevent similar issues would include further decontamination of the RT-RPA set-up and including more non-template control reactions, spread throughout the tested tubes. With these strategies in place, false positives can be avoided and the test’s specificity could reach up to 100%.

We compared our results with other RPA-based SARS-CoV-2 rapid nucleic acid tests reported in the literature. The multi-gene RT-RPA assay reported by Wahed et al. [11] and validated with 36 clinical samples showed high clinical sensitivity and specificity, equal to 94% and 100%, respectively, with their RdRP gene target, but lower results were achieved with their E gene target (65% sensitivity and 77% specificity). Moreover, the different genes cannot be detected simultaneously in their setup. Lau et al. [12] SARS-CoV-2 RT-RPA assay targeting the N gene only was validated with 113 clinical samples showing high sensitivity and specificity (98% and 100%, respectively). Another assay using RT-RPA and lateral flow tests, also using a single N gene target, was 100% concordant with RT-PCR results when validated with 37 clinical samples [13]. The CRISPR/Cas13 and RT-RPA combined fluorescence assay developed by Patchsung et al., validated with 154 clinical samples, reported high sensitivity and specificity equal to 96% and 100% (all samples), respectively [15].

Our results, therefore, are comparable with published results of similar assays with a higher number of samples compared to most RT-RPA studies. Importantly, our assay can detect two gene targets simultaneously thanks to the use of two distinct fluorophores and a multi-channel fluorescence reader allowing for further multiplexing. Moreover, we included clinically relevant respiratory pathogens as SARS-CoV-2 negative samples. We also tested for a range of Cq values with clinical relevance and, notably, we screened many SARS-CoV-2 lineages. Data on the robustness of RT-RPA assays when testing different SARS-CoV-2 variants is lacking in the literature.

Cq values may be cautiously used as a surrogate for viral loads and absolute quantification can be achieved using the standard curve method [37]. Therefore, low Cq values are equivalent to high viral loads and inversely. The significant correlation found between Cq and the TT revealed this method may be useful in clinical settings, for instance, the viral load of COVID-19 patients was shown to have direct clinical relevance to the risk of transmission [38]. Thus, when the TT is low, it will likely correlate with a high viral load, and a high TT will correlate with a low viral load. It is worth noting that the clinical samples used here were tested using an RT-PCR method targeting the N gene while the RT-RPA targeted two different genes (E and RdRP). This may have had an impact on the correlation reported, increasing the bias and variability of our results. Further work is planned to investigate the use of TT to draw standard curves for viral loads quantification using RT-RPA for both E and RdRP genes, for example using quantified quality control calibrators.

In our study of variants, we note that four VOCs known to date were detectable. The Gamma variant could not be studied herein due to unavailable clinical samples; Gamma prevalence was low in patients in the UK. We demonstrated high sensitivity for VOCs detection, proving that the assay targets conserved regions of the SARS-CoV-2 genome. Demonstrating pan-SARS-CoV-2 detection is crucial as new variants will keep emerging unpredictably and they may cause false negatives [39]. Molecular assays with a single target or targeting more variable regions are unlikely to be ‘variant-proof’ and may experience target failure, as this was particularly the case with the heavily mutated Omicron variant [26]. We confirmed that this is not the case with our assay, as the selected E and RdRP gene targets were not affected by Omicron mutations.

Future work will look at testing non-extracted samples which could establish an even faster and simpler diagnostic platform, for instance using unprocessed saliva swabs.

## Figures and Tables

**Table 1:** Clinical validation of the RT-RPA assay.

		RT-PCR				
		Positive	Negative			
**RT-RPA**	Positive	53	1	*N*	Sensitivity (95% CI)	Specificity (95% CI)
	Negative	2	35	91	96% (86–99)	97% (84–100)

*N*: number of samples; 95% CI: 95% confidence interval.

**Table 2:** VOCs detection with the RT-RPA assay.

VOCs Detection				
VOC	Alpha (B.1.1.7)	Beta (B.1.351)	Delta (B.1.617.2)	Omicron (B.1.1.529)
Sensitivity (95% CI)	87% (58–98)	100% (31–100)	100% (77–100)	100% (69–100)
Number of samples	15	3	14	10

95% CI: 95% confidence interval.

## Data Availability

The raw data that support the findings of this study are available from the corresponding authors, R.A.M. and E.N., upon reasonable request.

## Supplementary Materials

The following are available online at https://www.mdpi.com/article/10.3390/diagnostics12051263/s1, Supplementary Table S1: Clinical validation panel (91 clinical samples), Supplementary Table S2: Confirmation of presumptive positive results by real-time RT-PCR (N1 and E gene), Supplementary Table S3: Correlation between Cq (from RT-PCR N gene) and time to threshold (from RT-RPA E and RdRP gene), Supplementary Figure S1: Alignment of SARS-CoV-2 and HCoV-NL63 genomes, Supplementary Figure S2: Repeats of HCoV-NL63 (sample 16).
