# Supplementary material for: Clinical Validation of a Rapid Variant-Proof RT-RPA Assay for the Detection of SARS-CoV-2
Source: Diagnostics (Basel). 2022 May 19;12(5):1263. doi: 10.3390/diagnostics12051263 (PMC9141210; doi:10.3390/diagnostics12051263)
Supplement: Supplementary file 1 [file diagnostics-12-01263-s001.zip › diagnostics-1701357-supplementary.pdf]

Supplementary Information

Clinical validation of a rapid variant-proof RT-RPA assay for the detection of SARS-CoV-2

Dounia Cherkaoui[1,2,a], Judith Heaney[3,a], Da Huang[1], Matthew Byott[3,4], Benjamin S. Miller[1], Eleni Nastouli[3,4,*] & Rachel A. McKendry[1,2,*]

[1] London Centre for Nanotechnology, University College London, London WC1E 6BT, UK.

[2] Division of Medicine, University College London, London WC1E 6BT, UK.

[3] Advanced Pathogen Diagnostics Unit, University College London Hospitals (UCLH) NHS Trust, London NW1 2BU, UK.

[4] Infection, Immunity and Inflammation Department, Great Ormond Street Institute for Child Health (ICH), UCL, London WC1N 1EH, UK.

[a] These authors contributed equally to this work

* Corresponding authors: r.a.mckendry@ucl.ac.uk, e.nastouli@ucl.ac.uk

Supplementary Table S1. Clinical validation panel (91 clinical samples)

Samples	Pathogen(s)	Lineage	Cq	RT-RPA result	TT E|RdRP (sec)	Comparative result
1	SARS-CoV-2	B.1.1.7	31.1	Positive	655|689	TP
2	SARS-CoV-2	B.1.1.7	20.1	Positive	417|450	TP
3	Flu A			Negative		TN
4	SARS-CoV-2	B.1.1.216	30.0	Positive	417|450	TP
5	SARS-CoV-2	B.1.1.7	32.0	Negative		FN
6	SARS-CoV-2	B.1.1.7	30.0	Positive	554|588	TP
7	SARS-CoV-2	B.1.1.7	31.0	Positive	588|621	TP
8	SARS-CoV-2	B.1.1.7	22.2	Positive	417|450	TP
9	RSV			Negative		TN
10	RSV			Negative		TN
11	Enterovirus			Negative		TN
12	RSV			Negative		TN
13	SARS-CoV-2	B.1.1.307	27.3	Positive	485|554	TP
14	SARS-CoV-2	B.1.177.18	29.6	Presumptive positive	1065|X	TP
15	SARS-CoV-2	B.1.1.7	25.0	Positive	450|519	TP
16	HCoV-NL63			Positive	822|890	FP
17	SARS-CoV-2	B.1.1.311	32.0	Positive	855|890	TP
18	HCoV-HKU1			Negative		TN
19	SARS-CoV-2	B.1.1.7	24.4	Positive	413|446	TP
20	Flu A			Negative		TN
21	Flu A			Negative		TN
22	SARS-CoV-2	B.1.1.7	37.3	Negative		FN
23	Flu B			Negative		TN
24	Flu B			Negative		TN
25	Flu A			Negative		TN
26	Rhinovirus			Negative		TN
27	HCoV-229E			Negative		TN
28	Flu A			Negative		TN
29	Flu A			Negative		TN
30	SARS-CoV-2	B.1.1.7	24.3	Positive	480|514	TP
31	SARS-CoV-2	B.1.177.6	33.0	Positive	406|438	TP
32	SARS-CoV-2	B.1.1.7	23.8	Positive	406|438	TP
33	HCoV-OC43			Negative		TN
34	Flu B			Negative		TN
35	SARS-CoV-2	B.1.1.7	33.9	Positive	624|654	TP
36	SARS-CoV-2	B.1.1.216	31.3	Positive	438|468	TP
37	Flu A			Negative		TN
38	Flu A			Negative		TN

39	SARS-CoV-2	B.1.177	24.9	Positive	1088|872	TP
40	SARS-CoV-2	B.1.177	20.7	Positive	375|406	TP
41	SARS-CoV-2	B.1.177	21.4	Positive	345|375	TP
42	SARS-CoV-2	B.1.1.7	21.8	Positive	468|499	TP
43	SARS-CoV-2	B.1.1.29	35.0	Positive	685|717	TP
44	SARS-CoV-2	B.1.351	29.6	Positive	450|655	TP
45	SARS-CoV-2	B.1.617.2	16.1	Positive	214|272	TP
46	SARS-CoV-2	B.1.617.2	24.2	Positive	714|596	TP
47	SARS-CoV-2	B.1.1.7	28.9	Positive	508|567	TP
48	Flu A			Negative		TN
49	SARS-CoV-2	B.1.1.529	24.5	Positive	508|508	TP
50	SARS-CoV-2	B.1.617.2	29.3	Positive	450|479	TP
51	SARS-CoV-2	B.1.617.2	27.9	Positive	802|714	TP
52	SARS-CoV-2	B.1.1.529	22.7	Positive	390|450	TP
53	Flu A			Negative		TN
54	SARS-CoV-2	B.1.1.529	24.5	Positive	390|450	TP
55	SARS-CoV-2	B.1.351	24.4	Positive	567|538	TP
56	RSV, Adenovirus, Enterovirus			Negative		TN
57	RSV			Negative		TN
58	SARS-CoV-2	B.1.617.2	27.5	Positive	532|532	TP
59	SARS-CoV-2	B.1.617.2	30.4	Positive	503|532	TP
60	SARS-CoV-2	B.1.351	26.8	Positive	768|798	TP
61	SARS-CoV-2	B.1.1.529	27.1	Positive	445|503	TP
62	Rhinovirus			Negative		TN
63	Rhinovirus, Enterovirus			Negative		TN
64	SARS-CoV-2	B.1.1.529	30.7	Positive	621|679	TP
65	SARS-CoV-2	B.1.1.529	22.1	Positive	327|415	TP
66	Flu A			Presumptive positive	768|X	TN
67	SARS-CoV-2	B.1.177	29.7	Positive	269|494	TP
68	SARS-CoV-2	B.1.617.2	16.3	Positive	397|397	TP
69	SARS-CoV-2	B.1.617.2	19.2	Positive	397|430	TP
70	SARS-CoV-2	B.1.617.2	23.3	Positive	430|463	TP
71	SARS-CoV-2	B.1.617.2	21.1	Positive	430|430	TP
72	SARS-CoV-2	B.1.1.529	30.3	Positive	624|655	TP
73	RSV			Negative		TN
74	RSV			Negative		TN
75	SARS-CoV-2	B.1.617.2	17.5	Positive	397|463	TP
76	HCoV			Presumptive positive	494|X	TN
77	RSV			Negative		TN
78	SARS-CoV-2	B.1.1.529	31.3	Positive	494|559	TP

79	Flu A			Negative		TN
80	SARS-CoV-2	B.1.1.529	31.4	Positive	909|998	TP
81	SARS-CoV-2	B.1.617.2	13.6	Positive	384|442	TP
82	SARS-CoV-2	B.1.177	28.8	Presumptive positive	X|851	TP
83	Rhinovirus			Negative		TN
84	SARS-CoV-2	B.1.1.294	33.0	Positive	822|793	TP
85	RSV			Negative		TN
86	SARS-CoV-2	B.1.617.2	16.7	Positive	471|471	TP
87	Flu A			Negative		TN
88	SARS-CoV-2	B.1.1.7	34.9	Positive	822|822	TP
89	HCoV			Negative		TN
90	SARS-CoV-2	B.1.1.529	21.7	Positive	442|471	TP
91	SARS-CoV-2	B.1.617.2	29.2	Positive	791|822	TP

SARS-CoV-2: severe acute respiratory syndrome coronavirus 2; HCoV: human coronavirus; RSV: respiratory syncytial virus; Flu: influenza; Cq: quantification cycle; TT: time to threshold; X: used instead of TT for a gene that was not detected by RT-RPA; TP: true positive; TN: true negative; FP: false positive; FN: false negative.

Supplementary Table S2. Confirmation of presumptive positive results by real-time RT-PCR (N1 and E gene)

Sample ID	Confirmatory real-time RT-PCR Cq values
	CDC N1 gene
14	33.22
67	Undetermined
78	Undetermined
84	30.092

Supplementary Table S3. Correlation between Cq (from RT-PCR N gene) and time to threshold (from RT-RPA E and RdRP gene)

Pearson r	Cq vs. E gene (FAM)	Cq vs. RdRP gene (HEX)
r	0.511	0.629
95% confidence interval	0.277 to 0.688	0.430 to 0.770
R squared	0.261	0.396
P (two-tailed)	0.0001	<0.0001
Significant? (alpha = 0.05)	Yes	Yes

```
E gene
                                           E gene forward primer
SARS-CoV-2      --TGCTTTCGTGGTATTCTTGCTAGTTACACTAGCCATCCTTACTGCGCTTCGATTGTGT
HCoV-NL63       TGTTGTTTCGATTCATACATGTTGGCTATTATGCCTATCTCTATAAAAATT---TTTCAT


SARS-CoV-2      GCGTACTGCTGCAATATTGTTAACGTGAGTCTTGTAAAACCTTCTTTTTACGTTTACTCT
HCoV-NL63       TTGTTTTGTT-CAATGTTACTAAACTATGCTTCGT-----------------TTCAGGCA

                      E gene exo probe
SARS-CoV-2      CGTGTTAAAAATCTGAATTCTTCTAGAGTTCCTGATCTTCTGGTCTAAACGAACTAAATA
HCoV-NL63       AGTGTTGGTATCTTGAA---------------CAATCATTT-----------------TA

                           E gene reverse primer
SARS-CoV-2      TTATATTAGTTTTTCTGTTTGGAACTTTAATTTTAGCCATGGCAGATTCCAACGGTACTA
HCoV-NL63       TGAAAATCGTTTTGCTG---------CTATTTATGGTGGTGAC------------CACTA


RdRP gene
                         RdRP gene forward primer
SARS-CoV-2      GCTAATGAGTGTGCTCAAGTATTGAGTGAAATGGTCATGTGTGGCGGTTCACTATATGTT
HCoV-NL63       GGTAATGAGTTGGCACAAGTTTTAACAGAAGTTGTTTATTCTAATGGTGGTTTTTATTTT


SARS-CoV-2      AAACCAGGTGGAACCTCATCAGGAGATGCCACAACTGCTTATGCTAATAGTGTTTTTAAC
HCoV-NL63       AAGCCAGGTGGTACGACTTCTGGTGACGCTAGTACAGCTTATGCTAATTCTATTTTTAAC

                                                 RdRP gene exo probe
SARS-CoV-2      ATTTGTCAAGCTGTCACGGCCAATGTTAATG------------------CACTTTTATCT
HCoV-NL63       ATTTTTCAAGCCGTGAGTTCTAACATTAACAGGTTGCTTAGTGTCCCATCAGATTCATGT

                                              RdRP gene reverse primer
SARS-CoV-2      ACTGATGGTAACAAAATTGCCGATAAGTATGTCCGCAATTTACAACACAGACTTTATGAG
HCoV-NL63       AATAATGTTA------------------ATGTTAGGGATCTACAACGACGTCTGTATGAT
```

Supplementary Figure S1. Alignment of SARS-CoV-2 and HCoV-NL63 genomes. Sequence alignment of SARS-CoV-2 (GenBank accession number NC_045512.2) and HCoV-NL63 (GenBank accession number NC_005831) genomes using MUSCLE tool (https://www.ebi.ac.uk/Tools/msa/muscle/). The percent identity for primers and probes for both E and RdRP genes were calculated: E gene forward primer 40% identity, E gene reverse primer 43% identity, E gene exo probe 31% identity, RdRP gene forward primer 67% identity, RdRP gene reverse primer 43% identity and RdRP gene exo probe 63% identity.

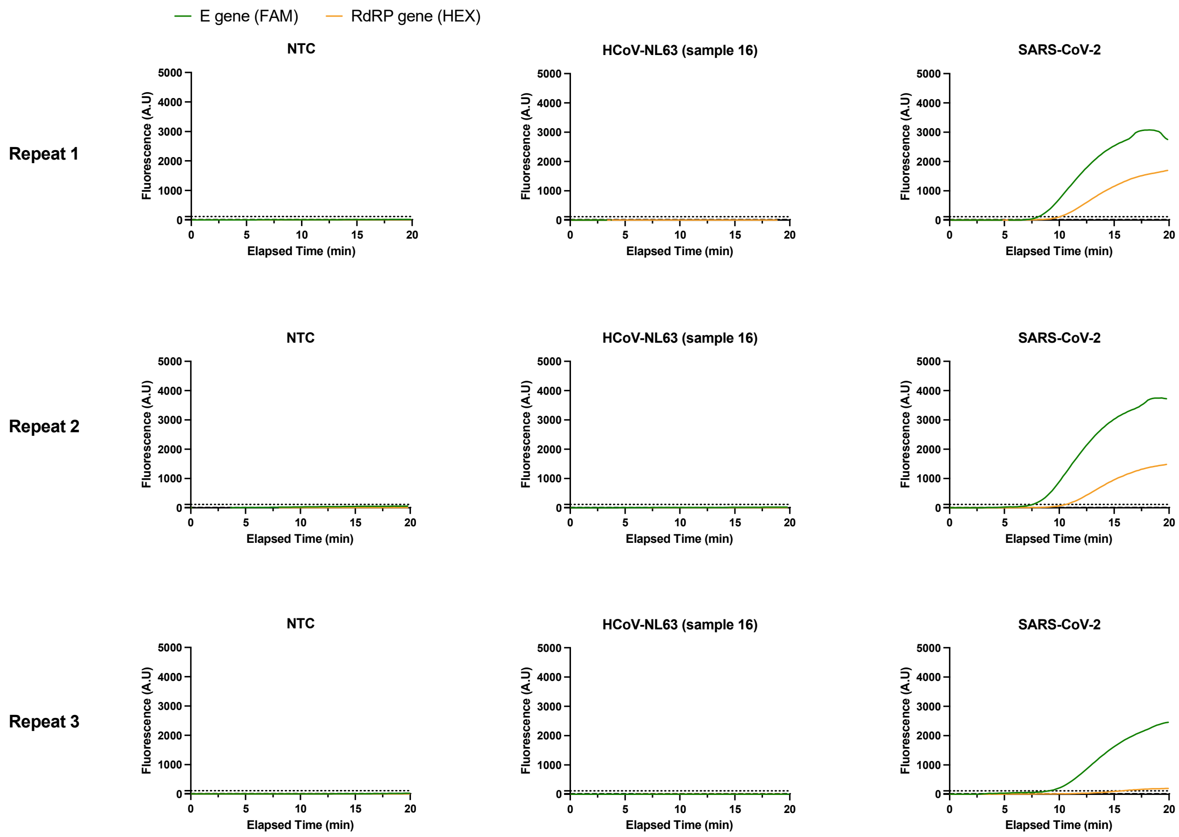

Supplementary Figure S2. Repeats of HCoV-NL63 (sample 16)

NTC: non-template control; HCoV-NL63: human coronavirus NL63
